# Beyond the Device: A Digital Infrastructure Framework for Sustainable Point-of-Care Diagnostic Services in Low-Resource and Infrastructure-Constrained Settings

**DOI:** 10.3390/diagnostics16162517

**Published:** 2026-08-10

**Authors:** Ingeborg M. Rocker, Kabir S. Patel

**Affiliations:** I2CE Lab, Georgia Institute of Technology, Atlanta, GA 30332, USA; kpatel721@gatech.edu

**Keywords:** point-of-care diagnostics, low-resource settings, digital health infrastructure, implementation science, design controls

## Abstract

Point-of-care and near-patient diagnostics can shorten the time between testing and clinical or public-health action, but a technically effective test does not by itself create a sustainable diagnostic service. Evidence from diagnostic-access research, digitally connected point-of-care testing, human-centered design, and implementation science indicates that diagnostic technologies must be developed as components of wider service systems encompassing workflows, quality assurance, data governance, maintenance, supply chains, user trust, and linkage to care. Drawing on these bodies of literature, this Perspective introduces the I2Med digital infrastructure framework for point-of-care diagnostics in low-resource and infrastructure-constrained settings. These settings are defined functionally as clinical or public-health environments in which resource or infrastructure constraints can disrupt one or more stages between testing and appropriate action. The I2Med framework integrates six domains: (1) access and context of use; (2) the result-to-action diagnostic workflow; (3) digital interfaces and data governance; (4) quality, risk, usability, and design-control alignment; (5) sustainability, maintenance, and supply continuity; and (6) equity, trust, and community accountability. Operational tools—including a setting typology, a domain-to-artifact matrix, an evidence-to-scale maturity ladder, and a hypothetical application vignette—illustrate how the framework can guide development and implementation planning. The I2Med framework’s central contribution is to reposition the diagnostic device as one component of an interconnected service system and to provide academic, translational, and early-stage development teams with a common structure for identifying implementation dependencies, evidence gaps, and responsibilities. As a conceptual framework, it requires prospective application and evaluation across diverse settings before its utility and transferability can be established.

## 1. Introduction

Point-of-care testing can bring diagnostic information closer to the patient and shorten the interval between a clinical question and a decision. Its value, however, lies not in speed alone but in delivering an interpretable, reliable, trusted, and actionable result within the setting in which a care or public-health decision must be made. A near-patient test that produces data without a defined result-to-action pathway may shorten turnaround time while leaving patients, clinicians, community health workers, or public-health programs uncertain about what should happen next.

This distinction is especially important in low-resource and infrastructure-constrained settings. In this manuscript, these terms describe heterogeneous care or public-health environments in which constraints on personnel, utilities, connectivity, procurement, maintenance, referral, financing, governance, or communication can interrupt the pathway from testing to action. They are not intended as deficit labels or as proxies for geography or national income. Such settings may include rural clinics, community health programs, mobile or ambulance-based services, disaster-response environments, home and self-testing contexts, small hospitals, primary-care facilities without rapid laboratory access, and health systems in which recurrent support is fragile. Because these constraints interact, failure at one point can compromise the entire diagnostic service.

Digital-health guidance cautions that digital interventions should strengthen health systems rather than operate as isolated technological fixes [1,2,3]. The peer-reviewed diagnostic-access and point-of-care literature likewise identifies affordability, usability, workflow, quality assurance, supply, sustainability, and linkage to care as determinants of access alongside analytical performance [4,5,6,7,8,9]. Studies of mobile-connected, wearable, and digitally enabled diagnostics further show that connectivity introduces dependencies on interfaces, data stewardship, interoperability, maintenance, and user trust [10,11,12,13,14].

Implementation frameworks and complex-intervention guidance, including NASSS, the updated CFIR, the updated MRC complex-interventions framework, RE-AIM extensions, and practical recommendations for framework use, explain why adoption, scale-up, spread, and sustainability depend on context, workflow, resources, leadership, user perception, maintenance, equity, and fit with the wider service environment [15,16,17,18,19]. However, these frameworks do not by themselves provide a diagnostic-specific bridge from implementation constructs to the concrete artifacts used in early development and design control. Such a bridge should connect use-context and workflow maps with data-flow diagrams, usability and risk plans, maintenance assumptions, evidence thresholds, and claim governance.

The problem addressed in this paper is therefore not simply technical. It is also an implementation and communication problem. Early-stage diagnostic teams often need to publish, apply for grants, recruit partners, and discuss implementation plans before device-specific intellectual-property protection, performance validation, or deployment evidence is complete. Silence can slow collaboration; overdisclosure can compromise patent strategy; and overclaiming can damage trust and create unrealistic expectations. The communication challenge is to be operationally specific without disclosing protected implementation details or implying evidence that does not yet exist.

We use “non-enabling disclosure” narrowly to describe communication that explains the problem, use context, workflow, infrastructure assumptions, evidence boundaries, and responsible claims while withholding legitimately protected implementation details. This principle must not be used to obscure methods, suppress unfavorable findings, or avoid the information required for scientific appraisal, ethical review, regulatory evaluation, or compliance with journal reporting standards.

This Perspective proposes the I2Med digital infrastructure framework for sustainable point-of-care diagnostic services in low-resource and infrastructure-constrained settings. Its six domains are: (1) access and context of use; (2) the result-to-action diagnostic workflow; (3) digital interfaces and data governance; (4) quality, risk, usability, and design-control alignment; (5) sustainability, maintenance, and supply continuity; and (6) equity, trust, and community accountability. The I2Med framework reframes decentralized diagnostics as service systems, maps these domains to actionable development artifacts and evidence thresholds, and supports stage-appropriate claims without requiring disclosure of protected technical details. It is intended as a planning and communication structure—not as a substitute for analytical or clinical validation, regulatory review, implementation evaluation, or prospective testing across diverse settings. We refer to this planning structure as the I2Med framework, using the name to identify the six-domain planning and communication framework rather than a specific device, assay, software product, or clinical service.

Table 1 defines the principal terms used throughout the Perspective. Table 2 positions the proposed framework relative to established digital-health, diagnostic-access, implementation-science, and medical-device guidance.

## 2. Scope and Framework-Development Approach

This manuscript is a Perspective and conceptual-framework article, not a systematic review, clinical validation study, regulatory submission, or product-performance report. It develops a practical framework for planning and communicating the digital and implementation infrastructure required to support point-of-care diagnostic services. The I2Med framework is intended for academic teams, translational researchers, technology-transfer offices, early-stage companies, funders, journal reviewers, and implementation partners who need to think beyond the device while making evidence-based, stage-appropriate claims and protecting legitimately confidential intellectual property.

The I2Med framework was developed through an iterative, targeted narrative synthesis rather than a systematic or scoping review. The literature search focused on peer-reviewed publications from January 2016 through July 2026, with older sources retained when they provided foundational concepts or essential regulatory, standards, or design-control context. Searches were updated through July 2026.

Searches were conducted in PubMed (National Library of Medicine, Bethesda, MD, USA; https://pubmed.ncbi.nlm.nih.gov/) and Google Scholar (Google LLC, Mountain View, CA, USA; https://scholar.google.com/; accessed 1 August 2026).. Publisher and DOI pages were used to retrieve or verify publications; relevant regulatory-authority and standards-body websites were searched for normative guidance. Backward citation searching was conducted from key publications addressing point-of-care diagnostics, digital health, implementation science, and diagnostic access. Search concepts included point-of-care diagnostics, low-resource settings, digitally connected diagnostics, mobile-linked diagnostics, user experience, REASSURED diagnostics, implementation science, design controls, usability, cybersecurity, sustainability, maintenance, supply continuity, equity, intellectual-property-aware scientific communication, and claim governance.

Generative-AI tools were not used to select sources, interpret evidence, verify references, or generate empirical findings. During revision, OpenAI’s ChatGPT (GPT-5; OpenAI, San Francisco, CA, USA; https://chatgpt.com/) was used for language-editing and drafting suggestions under author supervision; all scientific content, citations, interpretations, and submission decisions remained the responsibility of the authors..

Sources were selected when they clarified implementation determinants, diagnostic operations, digital-health governance, medical-device risk or usability, software or cybersecurity controls, sustainability planning, or responsible communication. Selection was purposive and directed toward conceptual relevance rather than comprehensive coverage. No formal risk-of-bias assessment, meta-analysis, or quantitative evidence synthesis was undertaken. This approach is appropriate to the conceptual aims of a Perspective but does not meet PRISMA-ScR requirements and should not be interpreted as a comprehensive map of the literature.

Six knowledge streams informed I2Med framework development: (1) the peer-reviewed digital-health, mobile-linked, and diagnostic-access literature; (2) implementation science and complex-intervention frameworks addressing adoption, nonadoption, abandonment, scale-up, spread, sustainability, and equity; (3) point-of-care diagnostic operations, including quality control, invalid-result handling, training, maintenance, stock continuity, documentation, and referral; (4) medical-device risk management, human factors, software life-cycle processes, artificial-intelligence and clinical-decision-support considerations, and cybersecurity governance; (5) sustainability and supply-chain planning for consumables, power, packaging, logistics, inventory, and end-of-life handling; and (6) non-confidential translational project experience from a multidisciplinary university environment.

These streams served different evidentiary functions and were not treated as equivalent forms of evidence. Regulatory and standards guidance was used as a normative design-control reference, not as peer-reviewed evidence of implementation effectiveness. Internal project experience was used only to generate planning questions and identify candidate failure modes; it was not treated as empirical evidence of diagnostic performance, usability, clinical effectiveness, cost-effectiveness, or implementation outcomes.

**Figure 1:**
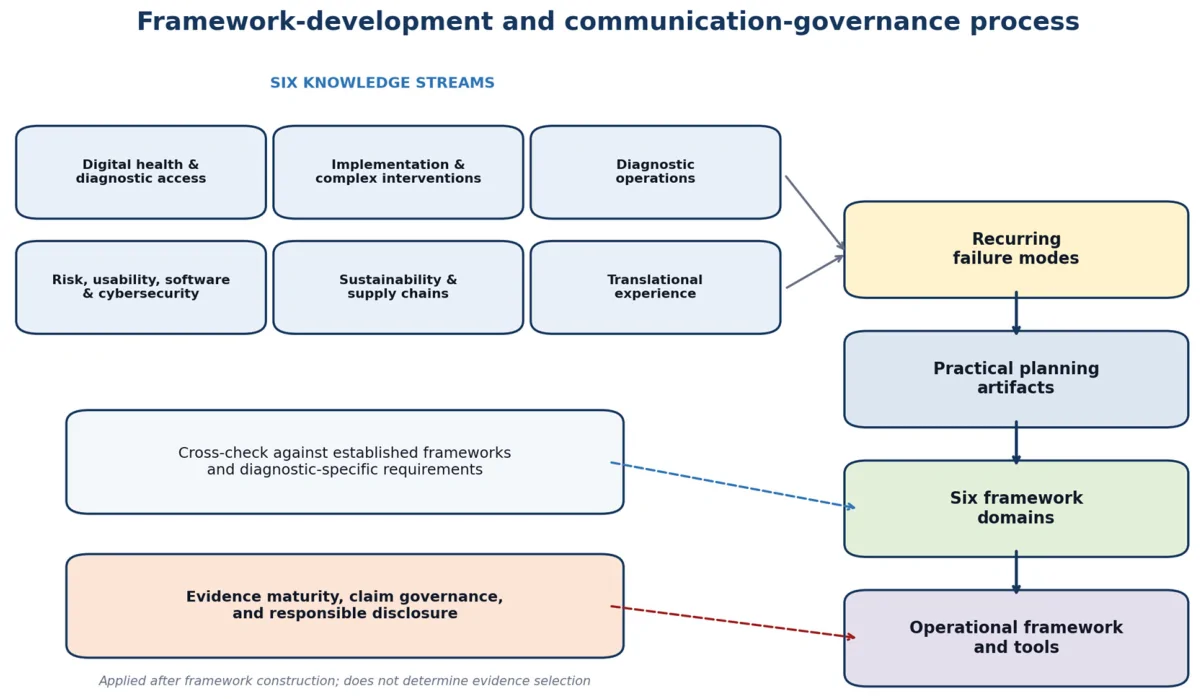
I2Med framework-development and communication-governance process. Six evidence and knowledge streams informed recurring diagnostic-service failure modes, which were mapped to planning artifacts and organized into six framework domains. Cross-checking against established frameworks informed domain boundaries. Evidence-maturity, claim-governance, and disclosure criteria were subsequently applied to support stage-appropriate communication.

The synthesis proceeded through four iterative steps. First, recurring failure modes in decentralized diagnostic planning were identified, including results without defined actions, digital interfaces without integrated workflows, prototypes without maintenance systems, claims unsupported by evidence, and access strategies without equity governance. Second, these failure modes were mapped to planning artifacts through which they could be identified and addressed during development. Third, the artifacts were grouped into six domains spanning access and context of use; the result-to-action diagnostic workflow; digital interfaces and data governance; quality, risk, usability, and design-control alignment; sustainability, maintenance, and supply continuity; and equity, trust, and community accountability. Fourth, the proposed domains and artifacts were compared with established digital-health, implementation, diagnostic, and design-control frameworks to identify areas of overlap and the diagnostic-specific contribution of the present framework. The framework-development and communication-governance process is summarized in Figure 1.

Disclosure and claim-governance criteria were applied only after the framework structure had been developed. These criteria governed how the framework and its examples were communicated; they did not determine which evidence was considered or how that evidence was interpreted.

This Perspective does not evaluate or make performance claims about a specific diagnostic product. Accordingly, it does not disclose proprietary device, assay, biomarker, algorithmic, software, or engineering implementations. No patient-level data are presented or used as evidence for the framework. These boundaries preserve protected technical work without withholding information required to understand, critique, or apply the conceptual framework. They do not supersede scientific-reporting standards, ethical obligations, journal policies, or regulatory requirements.

Several limitations follow from this approach. The purposive narrative synthesis may not capture all of the relevant literature and is subject to author selection and interpretation. The contribution of internal project experience is context-dependent and may not transfer to other development environments. Most importantly, the resulting framework is conceptual: its utility, usability, transferability, and effects on development or implementation decisions have not yet been established through prospective application across diverse settings.

## 3. Setting Typology for Diagnostic-Service Planning

Low-resource and infrastructure-constrained settings are not homogeneous and should not be treated as synonymous with low- and middle-income countries or with a generic underserved population. As defined above, these settings are characterized functionally by constraints that can interrupt one or more stages between testing and appropriate action. The relevant constraints may involve geography, infrastructure, financing, staffing, data capacity, procurement, maintenance, referral, communication, trust, emergency conditions, or continuity of support.

A diagnostic service model that is appropriate for a rural clinic may fail in a mobile screening program, whereas a home-testing model may create privacy or interpretation risks that differ from those in a staffed clinic. User-experience and social-science studies further indicate that the acceptability and use of point-of-care technologies are shaped by local care relationships, temporal demands, and health-system constraints [10,13]. The framework therefore begins with an explicit setting typology rather than a generic designation of “underserved”. Table 3 summarizes six setting archetypes, their characteristic access constraints and failure modes, and the minimum planning responses that should inform requirements, implementation plans, and public claims.

## 4. From Diagnostic Device to Diagnostic Service System

A point-of-care diagnostic is often described as a device, cartridge, reader, assay, software tool, or connected platform. In practice, however, the device operates as one component of a wider diagnostic service system. A diagnostic event includes a person with a clinical or public-health question, a setting with constraints, a user who performs or supervises testing, a specimen or other diagnostic input, a quality-control process, a result, an interpretation, a next step, documentation, and follow-up. Digital infrastructure should support the integrity and continuity of this pathway. It should not merely display the result.

The narrative synthesis identified four recurrent planning gaps that can separate an attractive diagnostic prototype from a deployable diagnostic service. The first is the result-to-action gap: the technology produces a result, but the care pathway does not specify what happens next. The second is the data-trust gap: data are collected or transmitted, but users do not understand who can access them, how they are protected, or how they will be used. The third is the maintenance-supply gap: the device works during demonstrations but fails when consumables, calibration, replacement parts, technical support, or waste handling are not funded or assigned to accountable owners. The fourth is the evidence-claim gap: design goals are repeated as if they were validated clinical, economic, equity, or implementation outcomes.

The framework is organized to make these gaps visible and support their systematic management. It does not assume that every early-stage team has complete evidence. Instead, it asks teams to make the current development and evidence stage, missing evidence, required artifacts, responsible owners, review points, and appropriate claims explicit. The framework does not guarantee that each gap will be resolved; it provides a structure through which unresolved dependencies can be identified before deployment or scale-up.

## 5. The Six-Domain I2Med Digital Infrastructure Framework

**Figure 2:**
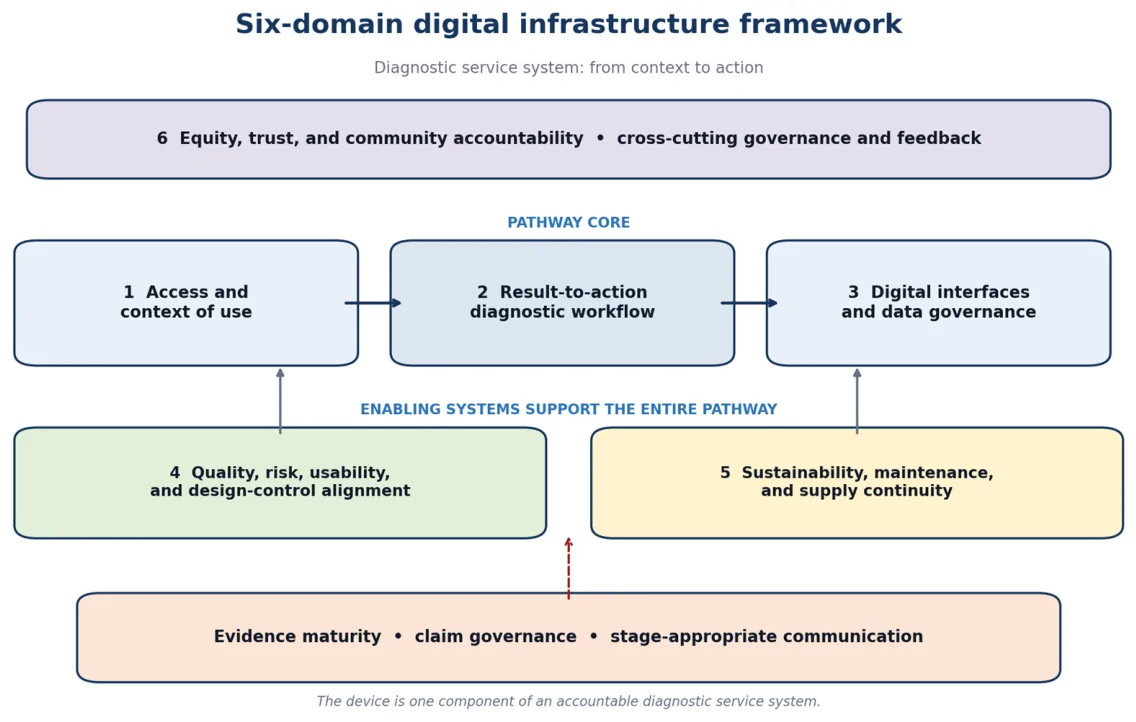
Six-domain I2Med digital infrastructure framework for sustainable point-of-care diagnostic services. The layered schematic distinguishes the diagnostic pathway core, enabling quality and service-continuity systems, and cross-cutting equity and community accountability. Evidence-maturity and claim-governance processes support stage-appropriate evaluation and communication across the framework. Colors distinguish framework domains and supporting governance layers; arrows indicate directional relationships among the pathway core, enabling systems, and cross-cutting accountability and communication processes.

Figure 2 summarizes the framework. Rather than presenting the domains as a linear sequence, the schematic distinguishes three interacting layers. Access and context of use, the result-to-action diagnostic workflow, and digital interfaces and data governance form the pathway core. Quality, risk, usability, and design-control alignment together with sustainability, maintenance, and supply continuity form enabling systems that support the entire pathway. Equity, trust, and community accountability operate across all domains. Evidence maturity and claim governance provide an accompanying accountability structure through which public claims are calibrated as evidence develops.

Table 4 operationalizes the framework by linking each domain to a primary planning artifact, a design-control or implementation output, and the principal risk that the artifact is intended to make visible.

### 5.1. Access and Context of Use

The access and context-of-use domain defines the people, places, constraints, and decisions that the diagnostic service is intended to support. A low-resource or infrastructure-constrained use context should not be described only as a population in need. It should specify the care setting, intended users, patient or public-health pathway, infrastructure assumptions, affordability constraints, language and literacy requirements, data environment, referral options, current alternatives, and what the proposed diagnostic service does not solve.

The framework asks teams to document whether testing is clinician-led, community-health-worker-led, caregiver-supported, patient-led, ambulance-based, or public-health-program-led, because each model creates different requirements for training, responsibility and oversight, result interpretation, documentation, privacy, and follow-up. The resulting use-context brief should distinguish assumptions supported by evidence from assumptions that still require field evaluation.

A useful access-context brief should include an explicit scope-boundary—or “does not solve”—statement. For example, a near-patient test may reduce the time to result but may not solve clinician shortages, medication affordability, transportation barriers after referral, or distrust of data collection. Naming what the diagnostic service does not solve prevents access claims from becoming broader than the evidence and reduces the risk of presenting the test as a stand-alone solution to structural inequity.

### 5.2. Result-to-Action Diagnostic Workflow

The workflow domain centers on the diagnostic decision rather than the digital output. A connected diagnostic service should define who initiates testing, who performs or supervises the test, who receives the result, who interprets it, the actions associated with each result category—including positive, negative, invalid, borderline, or clinically discordant results—and how repeat testing, escalation, referral, documentation, and follow-up are completed. The pathway should also specify responsibilities and contingencies for quality-control failures, expired consumables, device downtime, connectivity interruptions, and incomplete referral.

This domain distinguishes a dashboard from a care pathway. A dashboard that displays results is not equivalent to a workflow that produces action. For decentralized diagnostics, the more consequential design question is not merely, “Can the result be shown?” It is, “Can the right person understand the result, assess its quality and uncertainty, act on it within an authorized pathway, document the action, and close the loop?”

### 5.3. Digital Interface and Data Governance

The digital layer should be role-specific, privacy-aware, and accountable. Patients, community health workers, clinicians, program administrators, public-health teams, and maintenance personnel may each need different information from the same diagnostic event. A patient may need plain-language interpretation and next steps; a clinician may need quality-control status and clinical context; a program manager may need stockout and throughput indicators; a maintenance team may need device-health information; and a public-health unit may need appropriately governed, potentially de-identified or aggregated trends. Treating all users as one dashboard audience creates usability and privacy risks.

Data governance should be defined early, even when the first prototype is simple. At minimum, the team should specify what data are collected, why they are needed, who can access them, how consent or notice is handled, how long data are retained, how data are transmitted or stored, what happens offline, how exceptions are recorded, and how auditability is preserved. The plan should also identify data stewardship and accountability, applicable legal or institutional requirements, data-minimization principles, breach or incident response, and procedures for correcting erroneous records. Peer-reviewed digitally connected diagnostics studies identify connectivity, offline function, language, usability, data security, user support, and trust as implementation barriers or enablers [11,12,13]. Relevant guidance further indicates that remote data acquisition, clinical decision support, AI-enabled software, and cybersecurity controls must be evaluated within the intended sociotechnical and clinical system [20,21,22,23,24,25,26,27,28,29,30,31]. These distinct claims should be linked to the specific references that support them.

### 5.4. Quality, Risk, Usability, and Design-Control Alignment

The design-control domain translates implementation concerns into traceable requirements. Early-stage teams often treat workflow, usability, data governance, and sustainability as downstream deployment matters. The framework treats them as design inputs that should influence requirements, risk controls, verification plans, usability evaluation, software life-cycle planning, and evidence generation where applicable to the technology, development stage, jurisdiction, and intended use. This approach does not replace a product-specific regulatory or quality-management strategy and does not itself demonstrate compliance, but it helps teams avoid a common problem: building a technically promising prototype whose use conditions, operator assumptions, or maintenance requirements were never adequately defined.

For point-of-care diagnostics, design-control alignment should include the intended-use environment, intended users, foreseeable misuse, use-related hazards, user-interface risks, sample or input handling assumptions, quality-control status, invalid-result handling, data integrity, cybersecurity risks, training requirements, maintenance requirements, and traceability between user needs, design inputs, verification, validation, and applicable post-deployment monitoring and learning. Human factors and risk management are therefore not late-stage additions but integral inputs to whether results can be generated, interpreted, and acted upon safely.

Point-of-care testing management guidance and geospatial point-of-care planning likewise identify quality systems, operator training, emergency preparedness, population access, and maintenance infrastructure as operational prerequisites for trustworthy decentralized diagnostics [32,33]. The manuscript should distinguish whether these sources provide normative recommendations, empirical findings, or planning methods.

### 5.5. Sustainability, Maintenance, and Supply Continuity

Sustainability has environmental, operational, and financial dimensions. Environmental sustainability concerns device life, consumables, packaging, repairability, power use, cold-chain or storage needs where applicable, end-of-life handling, and the boundaries used to assess environmental burden. Operational sustainability concerns training, maintenance, troubleshooting, calibration where relevant, stock management, technical support, downtime response, replacement parts, and data storage. Financial sustainability concerns who pays, who procures, who bears recurring costs, whether the service is affordable to users and institutions, and how support continues after a pilot or grant ends.

A diagnostic deployment model can appear low-cost at launch while becoming unsustainable when consumables, staff time, maintenance, connectivity, data hosting, technical support, inventory management, distribution, human-resource capacity, or waste handling are included. Supply-chain studies of point-of-care diagnostic services in resource-constrained settings indicate that distribution, inventory management, and human-resource capacity can remain substantial access barriers even when procurement and quality-assurance processes are in place [34,35]. The framework therefore recommends a service-continuity plan that defines the total cost of service, responsible owners, recurrent resource requirements, fallback procedures, and sustainability boundaries before deployment or scale claims are made. In low-resource and infrastructure-constrained settings, the question is not only whether the device can work; it is whether the service model can remain reliable, affordable, maintainable, and appropriately supported over time.

### 5.6. Equity, Trust, and Community Accountability

Equity is not achieved simply because a diagnostic is placed in an underserved setting. Equity requires attention to who can access testing, who can understand results, who controls data, who is burdened by follow-up, who pays, who benefits, and who may be harmed by misuse or exclusion. Relevant design questions include language accessibility, disability access, health literacy, cultural trust, stigma, immigration or surveillance concerns, data-use transparency, community governance, and avoidance of punitive data uses. Teams should also examine whether benefits, burdens, errors, and opportunities for redress are distributed differently across population groups. Qualitative and stakeholder-engagement studies indicate that user experience, trust, local expertise, language, privacy, connectivity, and follow-up are not downstream adoption issues; they are part of the diagnostic intervention itself [10,11,12,13].

Trust is built before, during, and after deployment through transparent communication, limitation disclosure, respectful engagement, visible next steps, accountable referral pathways, and feedback and grievance mechanisms. A connected diagnostic system that produces technically correct results but leaves users confused about uncertainty, data use, or follow-up may be inequitable in practice. The framework therefore treats trust not as a marketing outcome but as a relational and governance requirement that must be supported by accountable processes and evaluated in context.

## 6. Evidence Maturity and Scale-Up Claims

Responsible scale-up requires evidence that is sufficient for the intended claim, population, setting, and decision. A concept-stage intervention can describe the access problem and intended design goals. It should not claim diagnostic performance, cost-effectiveness, clinical impact, environmental superiority, or scale readiness. As evidence grows, claims can become more specific but must remain bounded by the conditions under which that evidence was generated. Table 5 links each development stage to evidence needs and defensible public language.

The ladder is a communication and planning aid rather than a universal development sequence. Progress may be non-linear, and evidence may mature at different rates across analytical performance, clinical performance, usability, workflow, data governance, sustainability, equity, and implementation. A change in intended use, population, operator, setting, software, or support model may require reassessment rather than automatic progression.

To prevent the ladder from becoming a superficial checklist, each stage should have an evidence owner, an independent or appropriately authorized reviewer, a review date, a missing-evidence statement, and a decision rule for when specific claim language may be strengthened, retained, qualified, or withdrawn. For example, a stage-1 statement might say that a concept is designed to address a defined diagnostic-access barrier. A stage-3 statement might say that the system is prepared for controlled pilot evaluation with defined users and endpoints. At stage 5, authors may state only when supported that evidence supports scale-up under specified populations, settings, and support assumptions. This explicit evolution helps teams avoid sliding from design intent to validated impact without the evidence required to support that change. The stages are illustrative, and no stage label should substitute for the underlying evidence record.

## 7. Claim Governance and Scientifically Responsible Disclosure

As defined earlier, non-enabling publication is not vague publication and should not be used to avoid scientific reproducibility. Where a paper does not make product-specific performance claims, authors may describe the clinical or public-health need, use environment, workflow logic, framework, evidence plan, governance domains, sustainability boundaries, and implementation risks without disclosing legitimately confidential implementation details that are not necessary to evaluate those conceptual claims.

The disclosure boundary should be explicit and claim-dependent. The problem definition, framework-development approach, artifact types, evidence boundaries, limitations, and basis for every published claim must be reported. Product-specific architecture, assay, biomarker, sensor, algorithmic, or engineering details may remain outside scope only when they are unnecessary to appraise the claims being made and when their omission is consistent with applicable journal, ethical, regulatory, and legal requirements. If empirical performance, usability, or clinical-effect claims are made, the corresponding protocols, methods, evidence thresholds, results, uncertainties, and limitations must be disclosed at the level required for scientific review. Confidentiality or patent strategy cannot justify withholding evidence necessary to evaluate a published claim.

Claim governance is the operational mechanism that keeps communication aligned with evidence. Each external claim should be linked to an evidence level, intended audience, owner, source record or evidence version, review date, and approved language. This process is useful in manuscripts, abstracts, grant applications, conference posters, websites, partner memoranda, accelerator applications, and pitch decks. It also helps technology-transfer offices and collaborators identify language that could create intellectual-property, regulatory, or trust risks. Table 6 provides a practical claim-governance matrix. Approval responsibilities should be proportionate to the claim and should include relevant scientific, clinical, regulatory, ethical, community, or legal review where applicable.

The claim matrix should be used as a living review instrument. Teams should record the proposed claim, supporting evidence, missing evidence, intended audience, approver, and next review date. A claim can be strengthened only when new evidence directly supports the stronger language in the relevant context. Claims should also be qualified or withdrawn when evidence is contradicted, becomes outdated, or no longer applies after a change in intended use or setting. This governance step directly addresses the risk that the framework could be used as compliance language without the implementation work behind it.

## 8. Hypothetical, Non-Product-Specific Application Vignette

The following hypothetical pathway illustrates how the framework can be applied without revealing proprietary technical details. Consider an early-stage near-patient diagnostic service intended for a time-sensitive condition in a community or primary-care setting. The test category, specimen type, assay mechanism, device design, biomarker strategy, software implementation, and performance targets are intentionally left unspecified because the vignette does not evaluate a particular product. The vignette demonstrates how the framework organizes implementation questions; it does not establish product performance, usability, safety, effectiveness, or readiness for deployment.

The pathway begins with eligibility and the consent, authorization, or notice process appropriate to the intended use and jurisdiction. A trained user identifies whether the patient or participant fits the intended use context and explains what the test is intended to support, what it does not prove, what data are collected, and what follow-up may occur. The user then performs testing according to defined procedures. The system records operator role, test status, quality-control status, result category, and whether the result is valid, invalid, ambiguous, or requires repeat testing. The result is communicated through the appropriate role-specific interface. A concerning result triggers a defined referral or escalation step; a negative result triggers documented guidance and appropriate safety-netting consistent with the intended-use pathway; an invalid result triggers repeat testing, alternative testing, or referral according to protocol. The encounter is considered operationally complete only after documentation, patient communication, data handling, consumable use, waste handling, and any required follow-up have been completed or transferred to an accountable owner.

Using the framework changes the external communication. A conventional statement might say, “This low-cost diagnostic improves access and is easy to use in underserved communities.” The responsible version would say, “The project is designed to address a defined near-patient diagnostic-access barrier. The proposed service model specifies intended users, result categories, invalid-result handling, referral, data-governance, maintenance, and sustainability assumptions. Clinical performance, usability, cost, equity, and implementation outcomes require staged evaluation before claims about effectiveness or scale-up can be supported.” The second statement is longer, but it is more transparent and decision-relevant because it identifies both the intended value and the evidence gaps.

## 9. Practical Uses for Developers, Funders, Reviewers, and Implementation Partners

The framework can be used as a structured planning and review aid, grant-development structure, design-review template, technology-transfer screening tool, publication architecture, and partner-engagement map. Its practical value is that it converts broad implementation concerns into specific artifacts that can be assigned, reviewed, and updated. To keep the framework from becoming a superficial checklist, each artifact should identify an owner, current evidence level, unresolved assumptions, stakeholder-review status, and next decision point. Use of the framework does not demonstrate that an artifact is complete or that its underlying assumptions are valid.

Academic or translational diagnostic teams can use the framework to prevent the device from being treated as the whole intervention.

Medical-device or assay-development teams can connect user needs and risk controls to implementation assumptions.

Digital-platform teams can clarify role-specific interfaces, data-flow assumptions, access controls, and offline or exception handling.

Sustainability and life-cycle teams can define consumables, power, logistics, maintenance, end-of-life, and comparator boundaries before environmental or cost claims are made.

Funders and reviewers can assess whether implementation infrastructure is funded, not only prototype development.

Technology-transfer offices can support publication while preserving patent-sensitive implementation.

Community and implementation partners can see responsibilities, data use, referral, feedback, and grievance pathways before deployment.

Across these uses, the relevant output is not completion of the framework itself but a documented decision: proceed, revise, gather evidence, allocate resources, narrow a claim, or stop.

## 10. Evaluation Strategy and Future Research Agenda

The framework is designed to be evaluated. Future research should test whether use of the framework improves communication accuracy, implementation readiness, stakeholder understanding, decision quality, and selected implementation outcomes. Evaluation can occur through survey experiments, qualitative stakeholder interviews, design reviews, implementation pilots, document audits, and longitudinal analysis of claim changes over time. Comparative studies should specify the framework exposure, comparator, unit of analysis, follow-up period, and implementation context.

Seven candidate evaluation constructs warrant operational development and validation. Claim-evidence alignment measures whether public statements are supported by the evidence ledger. Stakeholder interpretability measures whether patients, clinicians, community workers, funders, and procurement stakeholders understand what has been proven, what remains uncertain, and what next step follows a result. Workflow actionability measures whether result categories trigger defined documentation, referral, repeat testing, or follow-up. Procurement readiness measures whether the package identifies buyer, payer, support model, training, maintenance, data-governance, and total-system cost assumptions. Sustainability readiness measures whether consumables, power, repair, replacement, waste, and life-cycle boundaries are explicit. Equity responsiveness measures whether language, accessibility, privacy, community input, and limitation disclosure are built into the deployment plan. Post-adoption learning quality measures whether feedback on usability, uptime, stockouts, result interpretation, referral completion, data quality, trust, and unintended consequences updates requirements and claims. These constructs are proposed domains of measurement, not validated instruments; future work should establish their reliability, validity, feasibility, and sensitivity to change.

The SWOT-style analysis in Table 7 summarizes practical barriers and opportunities. It is included to emphasize that the framework is not self-executing. Its value depends on whether teams fund, assign, review, and update the artifacts it identifies. Table 7 therefore belongs in the Discussion and should be interpreted as an author-generated implementation analysis rather than empirical evaluation.

The framework also has a future-facing role for next-generation decentralized diagnostics. Paper-based microfluidics, mobile-linked diagnostics, connected biosensors, wearables, AI-enabled decision support, and field-ready biomarker platforms can all increase reach, but they may also increase the importance of data governance, cybersecurity, user comprehension, algorithmic transparency, maintenance, supply continuity, and claim discipline [8,10,11,12,13,14,23,24]. The citations supporting technological capability, implementation experience, and governance requirements should be separated. Future work should test how the six domains need to be adapted for AI-enabled triage, multimodal sensing, offline-first architectures, federated or privacy-preserving data models, and integration with public-health surveillance while preserving patient trust and local accountability.

The framework generates five initial hypotheses for prospective study: (1) teams that define result-to-action workflows before pilot deployment will have fewer unresolved implementation assumptions than teams that define only technical performance goals; (2) evidence-linked claim language will produce more calibrated stakeholder trust than unqualified access, cost, usability, or equity claims; (3) a domain-to-artifact design-control matrix will improve grant and review credibility by making implementation work packages explicit; (4) framework-based, evidence-bounded communication will improve stakeholder understanding of both project value and uncertainty relative to unstructured project descriptions; and (5) maintenance, stock, and data-governance planning before deployment will be associated with fewer preventable service interruptions and unresolved ownership gaps in low-resource and infrastructure-constrained settings. Each hypothesis requires prespecified definitions, comparators, outcome measures, and study designs before it can be tested.

## 11. Boundary Conditions, Limitations, and Safeguards Against Misuse

This framework is most useful for early-stage, translational, and implementation-planning contexts in which a diagnostic concept is developing and the surrounding service infrastructure is still being specified. It is not a substitute for mature product-development documentation, formal quality-system procedures, regulatory submissions, clinical validation protocols, cybersecurity documentation, health-economic models, procurement contracts, or legal advice. Nor does use of the framework establish safety, effectiveness, regulatory compliance, implementation readiness, or suitability for scale-up.

The paper has limitations. It is a narrative Perspective rather than a systematic or scoping review, and the search process was not designed to provide exhaustive coverage, formal risk-of-bias assessment, or pooled estimates. The framework has not yet been prospectively validated in a deployed diagnostic program. It draws on the interdisciplinary literature and non-confidential translational experience, which may not capture all constraints in every geography, disease area, regulatory environment, or care model. Because the paper does not analyze a specific product, the hypothetical vignette cannot demonstrate product-level feasibility or implementation readiness; its purpose is limited to illustrating framework use. Regulatory and standards guidance is used here as planning context rather than empirical evidence of implementation effectiveness. The framework is oriented toward human clinical and public-health diagnostics; veterinary, environmental, agricultural, and non-human diagnostic applications may require adaptation because their users, governance structures, evidence expectations, and consequence pathways differ. The proposed domains and evaluation constructs may also require adaptation after stakeholder review and prospective application in settings not represented in the literature or the authors’ experience.

The framework can also be misused. Teams could adopt the language of infrastructure planning without actually performing the work; they could use non-enabling publication as a way to avoid evidence; or they could treat low-resource settings as generic test beds rather than accountable care environments. To reduce those risks, the framework proposes artifacts, owners, evidence thresholds, stakeholder-interpretability checks, and post-adoption claim review. A claim should not move up the maturity ladder unless the corresponding evidence and stakeholder-review criteria are met. These safeguards depend on transparent documentation, appropriately independent review, meaningful stakeholder participation, and willingness to narrow or withdraw claims when evidence is insufficient.

## 12. Conclusions

Sustainable and equitable point-of-care diagnostic services depend on more than technical device performance. They require a service system that links access and context of use, the result-to-action diagnostic workflow, digital interfaces and data governance, quality and risk controls, usability, maintenance, supply continuity, sustainability planning, equity safeguards, trust, and evidence-based decisions about implementation and scale-up. The I2Med framework presented here provides early-stage teams with a structured, evidence-bounded approach for planning and communicating that system without overstating readiness. Legitimately confidential technical details may remain outside scope only when they are unnecessary to appraise the claims being made and their omission complies with applicable scientific, ethical, journal, regulatory, and legal requirements.

The central message is simple: the test alone is not the complete intervention. A sustainable diagnostic service embeds the test and its result within a trustworthy, maintainable, data-governed, equitable, and actionable care or public-health pathway. When teams design the diagnostic, digital layer, care pathway, evidence plan, and deployment model together, they can identify implementation dependencies and evidence gaps before making claims about readiness or scale. Prospective application across diverse settings is now needed to determine whether the I2Med framework improves planning, communication, implementation decisions, and accountability.

## Figures and Tables

**Table 1:** Key terms used in this Perspective.

Term	Operational Definition	Guardrail
**Low-resource or infrastructure-constrained setting**	A heterogeneous care or public-health environment in which constraints can interrupt one or more steps between testing and appropriate action.	Not a deficit label or proxy for geography or national income; the relevant constraints must be specified.
**Non-enabling disclosure**	Communication that discloses use context, workflow, infrastructure assumptions, evidence boundaries, and claim logic while withholding legitimately protected implementation details.	Must not prevent scientific appraisal or override ethical, regulatory, reporting, or data-sharing requirements.
**Result-to-action gap**	The gap between producing a diagnostic result and ensuring that the right person can interpret, trust, document, and act on that result.	A dashboard or digital result display does not close this gap unless it is linked to defined responsibilities and care pathways.
**Claim governance**	A review process that links each public claim to the underlying evidence level, intended audience, owner, review date, and approved language.	Separates design goals and hypotheses from validated clinical, economic, equity, or scale outcomes.

**Table 2:** Relationship between established guidance and the contribution of the I2Med framework.

Evidence Base	What It Contributes	I2Med Framework Contribution
**WHO digital-health guidance and digital-intervention classifications [1,2,3]**	Health-system strengthening, governance, user needs, and classification of digital functions.	Links those principles to diagnostic result pathways, data flows, and maintenance assumptions.
**Global diagnostic-access and point-of-care literature [4,5,6,7,8,9]**	Defines access, affordability, deployability, and analytical-performance priorities.	Connects test performance to referral, documentation, quality systems, data governance, and continuity of service.
**Mobile-linked, digitally enabled, wearable, and biomarker-oriented POC literature [10,11,12,13,14]**	Demonstrates technical integration of sensing, mobile interfaces, and field use.	Surfaces the infrastructure dependencies and evidence thresholds that should precede scale claims.
**NASSS, CFIR, MRC, RE-AIM, and implementation/process-evaluation frameworks [15,16,17,18,19]**	Explains adoption, context, reach, implementation outcomes, sustainability, and evaluation.	Maps implementation constructs to diagnostic-development artifacts and accountable owners.
Medical-device, software, cybersecurity, usability, and risk guidance [20,21,22,23,24,25,26,27,28,29,30,31]	Sets expectations for human factors, software, cybersecurity, risk management, quality systems, and connected-device evaluation.	Aligns development evidence with stage-appropriate public claims while preserving required scientific and regulatory transparency.

**Table 3:** Setting typology and diagnostic-infrastructure implications.

Setting Archetype	Typical Access Constraint	Likely Failure Mode	Minimum Planning Response
**Rural or remote clinic**	Distance from central laboratory; limited staffing; transport delay.	Maintenance delays, stockouts, connectivity gaps, and limited referral options.	Define local training, maintenance, consumables, offline fallback, and escalation routes.
**Community outreach or mobile screening**	Patients may not present to fixed facilities; trust and follow-up may be fragile.	Result may not lead to confirmed care; records may be incomplete.	Map eligibility, consent or notice, result communication, referral completion, and documentation.
**Home or self-testing**	Convenience, stigma, transport burden, or limited clinic access.	Misinterpretation, privacy concerns, poor sample collection, or unreported results.	Design plain-language instructions, accessible support, data-use transparency, and next-step guidance.
**Ambulance, disaster, or crisis response**	Time-critical decisions outside standard facilities.	Harsh conditions, power limits, unreliable connectivity, and supply disruption.	Plan robustness, offline operation, rapid interpretation, chain of custody, and triage linkage.
**Small hospital or primary-care facility**	Limited immediate laboratory capability or specialist support.	Integration with existing workflow and records may be weak.	Specify operator roles, quality control, documentation, escalation, and electronic-record interface.
**Resource-constrained public-health system**	Budget limits, fragmented procurement, and competing priorities.	Pilot may end when donor or grant support ends.	Develop a total cost-of-service model, procurement pathway, maintenance responsibility, and sustainability boundary.

**Table 4:** Six I2Med framework domains, core artifacts, and principal risks.

Domain	Core Planning Artifact	Required Output	Principal Risk Controlled
**1 Access and context of use**	Use-context brief; patient/user journey map.	User needs; intended users; intended-use environment; access-barrier definition.	Technology is optimized for an imagined setting rather than deployment reality.
**2 Result-to-action workflow**	Diagnostic pathway map with result categories and escalation steps.	Workflow requirements; invalid-result protocol; referral and documentation requirements.	Data are generated but do not produce care action or follow-up.
**3 Digital interface and data governance**	Role-based interface map; data-flow diagram.	Data requirements; access control; consent/notice; audit trail; retention; interoperability assumptions.	The interface creates mistrust, privacy risk, or unusable information overload.
**4 Quality, risk, usability, and design-control alignment**	Risk-control log; usability plan; requirements traceability matrix.	Design inputs; verification plan; usability validation plan; software life-cycle controls.	An early prototype is mistaken for a controlled diagnostic system.
**5 Sustainability, maintenance, and supply continuity**	Service-continuity plan; sustainability boundary.	Maintenance; consumables; stockout response; service model; power; waste and life-cycle boundaries.	The pilot collapses because recurrent costs and support needs were not funded.
**6 Equity, trust, and community accountability**	Equity and trust plan; stakeholder feedback loop.	Accessible communication; community engagement; limitations; privacy expectations; grievance pathway.	Deployment fails to earn trust or worsens existing inequities.

**Table 5:** Evidence-to-scale maturity ladder for decentralized point-of-care diagnostics.

Stage	Evidence Focus	Defensible Public Claim	Claims Not Yet Supported
**0 Define context**	Access problem; setting; user roles; care pathway; stakeholder needs.	The project addresses a defined diagnostic-access barrier and has mapped the intended use context.	Improves outcomes; reduces disparities; lowers costs.
**1 Specify concept and requirements**	User needs; design inputs; risk hypotheses; data-governance assumptions; sustainability boundary.	The concept is designed to support a specified near-patient workflow and is planned for evaluation against defined requirements.	Clinically accurate; easy for anyone to use; ready for pilot deployment.
**2 Demonstrate feasibility**	Early feasibility and technical or requirements testing under defined conditions.	Preliminary feasibility or requirements testing has been conducted under defined conditions.	Validated; proven; equivalent to laboratory testing; deployment-ready.
**3 Establish controlled-pilot readiness**	Representative usability testing; quality procedures; workflow simulation; data-flow testing; maintenance planning.	The system is prepared for controlled pilot evaluation with defined users, settings, and endpoints.	Scalable; cost-saving; fully integrated; eliminates access gaps.
**4 Evaluate implementation**	Real-world workflow; training; uptime; stock; referral completion; data quality; user feedback; equity signals.	The deployment model is being evaluated for operational fit and implementation learning in a defined setting.	Generalizable across settings; environmentally superior; sustainable at scale.
**5 Support conditional scale-up**	Applicable analytical/clinical validation; implementation outcomes; health-economic, sustainability, and equity evidence.	Evidence supports scale-up under specified conditions, populations, settings, and support assumptions.	Universal solution; no infrastructure burden; guaranteed equity impact.

**Table 6:** Claim-governance matrix for early-stage point-of-care diagnostic infrastructure publications.

Claim Area	Evidence Required	Stage-Appropriate Language	Avoid Unless Supported
**Access**	Use-context evidence; feasibility plan.	Designed to address a defined diagnostic-access barrier in a specified near-patient setting.	Eliminates diagnostic inequity; solves access barriers.
**Workflow**	Result-to-action pathway; operator roles.	The intended workflow specifies result interpretation, invalid-result handling, referral, and documentation.	Seamlessly integrates into care; results automatically improve decisions.
**Performance**	Verification and applicable analytical and clinical validation.	Performance evaluation is planned or has been conducted, as applicable, against defined requirements and intended-use conditions.	Clinically accurate; validated; equivalent to central-laboratory testing without supporting evidence.
**Data governance and cybersecurity**	Data-flow map; privacy review; access controls; retention/audit assumptions; threat modeling.	Data collection, access, retention, auditability, and cybersecurity risks are defined for the intended connected setting.	Fully secure; privacy guaranteed; no cybersecurity risk.
**Sustainability and cost**	Total cost-of-service model; procurement pathway; maintenance and life-cycle boundaries.	Cost of use, consumables, maintenance, power, and end-of-life assumptions will be evaluated.	Low-cost deployment; environmentally superior; sustainable by design.
**Equity and trust**	Stakeholder engagement; accessibility review; limitations; community feedback pathway.	The deployment plan includes equity, accessibility, privacy, and stakeholder-engagement safeguards.	Empowers all communities; trusted by all users.

**Table 7:** SWOT-style implementation analysis for the proposed framework.

**STRENGTHS**	**WEAKNESSES**
Makes infrastructure assumptions explicit before deployment claims are made. Connects diagnostic, digital, workflow, quality, sustainability, and equity concerns.	Requires time, documentation discipline, and stakeholder access that early teams may not yet have. Does not replace validation, regulatory review, cybersecurity testing, or health-economic evaluation.
**OPPORTUNITIES**	**THREATS**
Can improve grant planning, design reviews, partner engagement, and publication quality. Can be adapted to mobile-linked, paper-based, wearable, and AI-enabled diagnostics.	May be treated as a superficial checklist if artifacts are not reviewed and updated. Could be misused to imply deployment readiness without funding the support system.

## Data Availability

No new data were created or analyzed in this study. Data sharing is not applicable to this article.

## References

[1] World Health Organization (2025). Global Strategy on Digital Health 2020–2027.

[2] World Health Organization (2019). WHO Guideline: Recommendations on Digital Interventions for Health System Strengthening.

[3] World Health Organization (2023). Classification of Digital Interventions, Services and Applications in Health: A Shared Language to Describe the Uses of Digital Technology.

[4] Fleming K.A., Horton S., Wilson M.L., Atun R., DeStigter K., Flanigan J., Sayed S., Adam P., Aguilar B., Andronikou S. (2021). The Lancet Commission on diagnostics: Transforming access to diagnostics. Lancet.

[5] Hayes K.L., Lieberman M. (2023). Considerations for the design and implementation of point-of-care technology for use in low- and middle-income countries. Nat. Rev. Methods Primers.

[6] Heidt B., Siqueira W.F., Eersels K., Diliën H., van Grinsven B., Fujiwara R.T., Cleij T.J. (2020). Point of care diagnostics in resource-limited settings: A review of the present and future of PoC in its most needed environment. Biosensors.

[7] Land K.J., Boeras D.I., Chen X.S., Ramsay A.R., Peeling R.W. (2019). REASSURED diagnostics to inform disease control strategies, strengthen health systems and improve patient outcomes. Nat. Microbiol..

[8] Nilghaz A., Guan L., Tan W., Shen W. (2016). Advances of paper-based microfluidics for diagnostics: The original motivation and current status. ACS Sens..

[9] Moetlhoa B., Maluleke K., Mathebula E.M., Kgarosi K., Nxele S.R., Lenonyane B., Mashamba-Thompson T. (2023). REASSURED diagnostics at point-of-care in sub-Saharan Africa: A scoping review. PLoS Glob. Public Health.

[10] Aborode A.T., Adesola R.O., Scott G.Y., Arthur-Hayford E., Otorkpa O.J., Kwaku S.D., Elebesunu E.E., Nibokun E.O., Aruorivwooghene I.J., Bakre A.A. (2025). Bringing lab to the field: Exploring innovations in point-of-care diagnostics for the rapid detection and management of tropical diseases in resource-limited settings. Adv. Biomark. Sci. Technol..

[11] Nxele S.R., Moetlhoa B., Dlangalala T., Maluleke K., Kgarosi K., Theberge A.B., Mashamba-Thompson T. (2024). Mobile-linked point-of-care diagnostics in community-based healthcare: A scoping review of user experiences. Arch. Public Health.

[12] Moetlhoa B., Nxele S.R., Maluleke K., Mathebula E., Marange M., Chilufya M., Dzinamarira T., Duah E., Dzobo M., Kekana M. (2024). Barriers and enablers for implementation of digital-linked diagnostics models at point-of-care in South Africa: Stakeholder engagement. BMC Health Serv. Res..

[13] Perkins J., Chandler C., Kelly A., Street A. (2024). The social lives of point-of-care tests in low- and middle-income countries: A meta-ethnography. Health Policy Plan..

[14] Kulkarni M.B., Rajagopal S., Prieto-Simon B., Pogue B.W. (2024). Recent advances in smart wearable sensors for continuous human health monitoring. Talanta.

[15] Greenhalgh T., Wherton J., Papoutsi C., Lynch J., Hughes G., A’Court C., Hinder S., Fahy N., Procter R., Shaw S. (2017). Beyond adoption: A new framework for theorizing and evaluating nonadoption, abandonment, and challenges to the scale-up, spread, and sustainability of health and care technologies. J. Med. Internet Res..

[16] Damschroder L.J., Reardon C.M., Widerquist M.A.O., Lowery J. (2022). The updated Consolidated Framework for Implementation Research based on user feedback. Implement. Sci..

[17] Skivington K., Matthews L., Simpson S.A., Craig P., Baird J., Blazeby J.M., Boyd K.A., Craig N., French D.P., McIntosh E. (2021). A new framework for developing and evaluating complex interventions: Update of Medical Research Council guidance. BMJ.

[18] Glasgow R.E., Harden S.M., Gaglio B., Rabin B., Smith M.L., Porter G.C., Ory M.G., Estabrooks P.A. (2019). RE-AIM planning and evaluation framework: Adapting to new science and practice with a 20-year review. Front. Public Health.

[19] Moullin J.C., Dickson K.S., Stadnick N.A., Albers B., Nilsen P., Broder-Fingert S., Mukasa B., Aarons G.A. (2020). Ten recommendations for using implementation frameworks in research and practice. Implement. Sci. Commun..

[20] U.S. Food and Drug Administration (2023). Digital Health Technologies for Remote Data Acquisition in Clinical Investigations: Guidance for Industry, Investigators, and Other Stakeholders.

[21] U.S. Food and Drug Administration (2016). Applying Human Factors and Usability Engineering to Medical Devices: Guidance for Industry and Food and Drug Administration Staff.

[22] U.S. Food and Drug Administration (2026). Content of Human Factors Information in Medical Device Marketing Submissions: Guidance for Industry and Food and Drug Administration Staff.

[23] U.S. Food and Drug Administration (2026). Cybersecurity in Medical Devices: Quality Management System Considerations and Content of Premarket Submissions: Guidance for Industry and Food and Drug Administration Staff.

[24] U.S. Food and Drug Administration (2026). Clinical Decision Support Software: Guidance for Industry and Food and Drug Administration Staff.

[25] U.S. Food and Drug Administration (2025). Marketing Submission Recommendations for a Predetermined Change Control Plan for Artificial Intelligence-Enabled Device Software Functions: Guidance for Industry and Food and Drug Administration Staff.

[26] (2019). Medical Devices—Application of Risk Management to Medical Devices.

[27] (2016). Medical Devices—Quality Management Systems—Requirements for Regulatory Purposes.

[28] (2015). Medical Device Software—Software Life Cycle Processes.

[29] (2020). Medical Devices—Part 1: Application of Usability Engineering to Medical Devices.

[30] International Medical Device Regulators Forum (2017). Software as a Medical Device (SaMD): Clinical Evaluation.

[31] International Medical Device Regulators Forum (2020). Principles and Practices for Medical Device Cybersecurity.

[32] Nichols J.H., Alter D., Chen Y., Isbell T.S., Jacobs E., Moore N., Shajani-Yi Z. (2020). AACC guidance document on management of point-of-care testing. J. Appl. Lab. Med..

[33] Kost G.J. (2019). Geospatial science and point-of-care testing: Creating solutions for population access, emergencies, outbreaks, and disasters. Front. Public Health.

[34] Maluleke K., Musekiwa A., Mashamba-Thompson T. (2023). Evaluating supply chain management of SARS-CoV-2 point-of-care diagnostic services in primary healthcare clinics in Mopani District, Limpopo Province, South Africa. PLoS ONE.

[35] Maluleke K., Musekiwa A., Nxele S., Moetlhoa B., Makena L., Nzuza N., Lenders A., Manentsa N., Maswanganyi T., Dlangalala T. (2024). Co-creation of a novel approach for improving supply chain management for SARS-CoV-2 point-of-care diagnostic services in Mopani District, Limpopo Province: Nominal group technique. Front. Public Health.

